# Supplementary material for: Hollow-Channel Paper Analytical Devices Supported Biofuel Cell-Based Self-Powered Molecularly Imprinted Polymer Sensor for Pesticide Detection
Source: Biosensors (Basel). 2022 Nov 5;12(11):974. doi: 10.3390/bios12110974 (PMC9687901; doi:10.3390/bios12110974)
Supplement: Supplementary file 1 [file biosensors-12-00974-s001.zip › biosensors-1972507-supplementary.pdf]

biosensors

MDPI

Supporting Information

Hollow-Channel Paper Analytical Devices Supported Biofuel Cell-Based Self-Powered Molecularly Imprinted Polymer Sensor for Pesticide Detection

Yanhu Wang [1,2,*], Huihui Shi [2], Jiantao Sun [3], Jianjian Xu [4], Mengchun Yang [1] and Jinghua Yu [2]

1 Shandong Analysis and Test Center, Qilu University of Technology (Shandong Academy of Sciences), Jinan 250014, China
2 School of Chemistry and Chemical Engineering, University of Jinan, Jinan 250022, China
3 Shandong Institute for Product Quality Inspection, Jinan 250102, China
4 Department of Food and Drug, Weihai Ocean Vocational College, Weihai 264300, China
* Correspondence: chm_wangyh@qlu.edu.cn

This supporting information includes:

Reagents and Materials.

Figures S1 to S8.

Scheme S1.

Reagents and Materials

All reagents were of analytical-reagent grade and directly used for the following experiments as supplied. Ultrapure water obtained from a Millipore water purification system (≥18.2 MΩ·cm, Milli-Q, Millipore) was used in all assays and solutions. Pyrrole (Py) and glucose oxidase (GOx) were purchased from Sigma-Aldrich. GOx-labeled 2,4-D (GOx-2,4-D) was obtained from Zhengzhou Biocell Biotechnology Co., Ltd. (China). Bilirubin oxidase (BOD) from Myrothe-cium verrucaria (E.C.1.10.3.2, 6 Umg-1) was purchased from Sigma. Carbon nanotubes (CNTs) were purchased from Shenzhen Nanotech. Port. Co. Ltd. (Shenzhen, China) without further purification. Tetrachloroauric acid ($HAuCl_4$) and sodium citrate was purchased from Sinopharm Chemical Reagent Co. Ltd. Whatman chromatography paper #1 (58.0 cm ×68.0 cm) was obtained from GE Healthcare Worldwide (Pudong Shanghai, China) and used with further adjustment of size (A4 size).

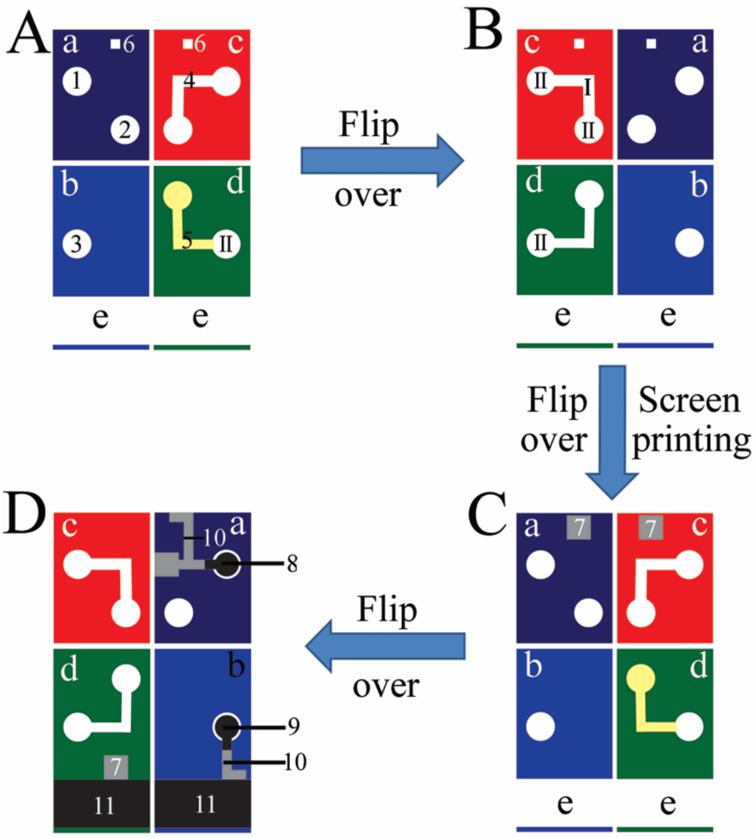

Figure S1. Schematic representation of this 3D-μ-MOBFCAD. (A) Wax patterns of this 3D-μ-MOBFCAD; (B) the reverse side of (A); (C) Right side of 3D-μ-MOBFCAD the after screen-printing of silver wire and carbon electrode; (D) the reverse side of (C). (a) cathodic tab; (b) anodic tab; (c) hollow-channel tab; (d) hemichannel tab; (e) unprinted paper for paper supercapacitor. (1) cathodic zone; (2) sample inlet; (3) anodic zone; (4) hollow-channel; (5) hemichannel; (6) via hole; (7) sliver pad; (8) screen-printed cathodic electrode (SPCE); (9) screen-printed anodic electrode (SPAE); (10) silver wire; (11) paper supercapacitor. (I, II) hollow area.

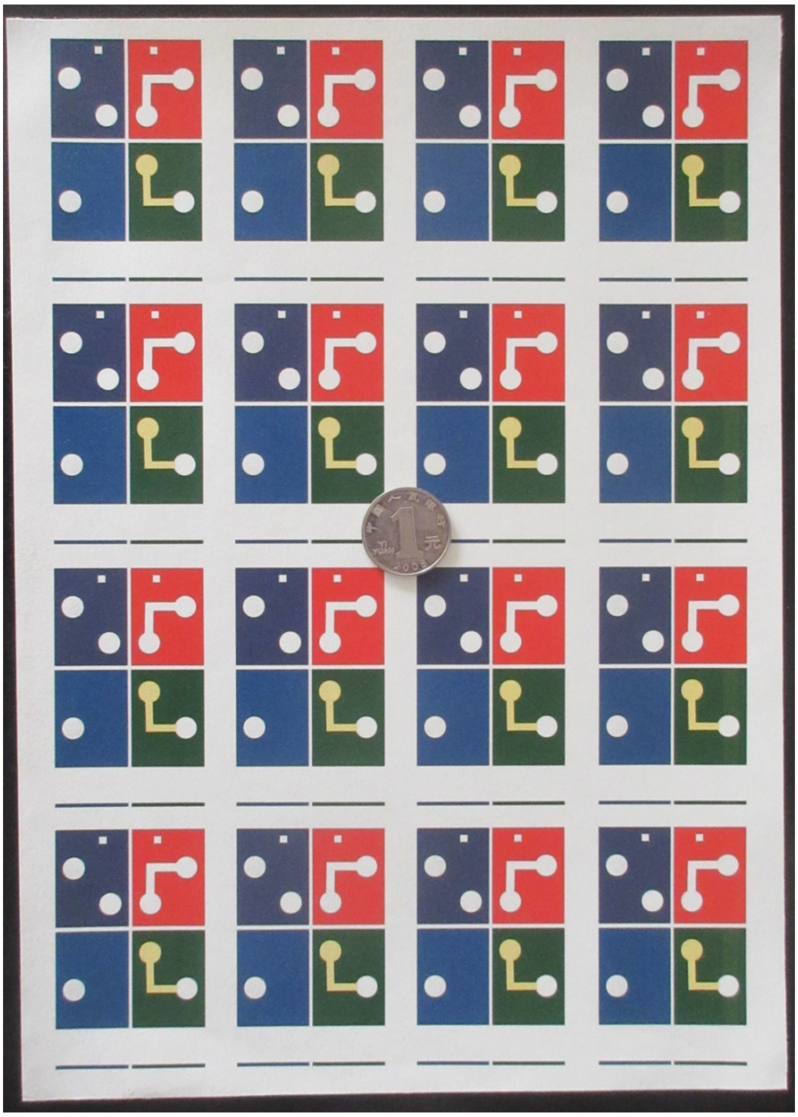

Figure S2. Wax-patterns of 3D-μ-MOBFCAD on a paper sheet (A4) before baking.

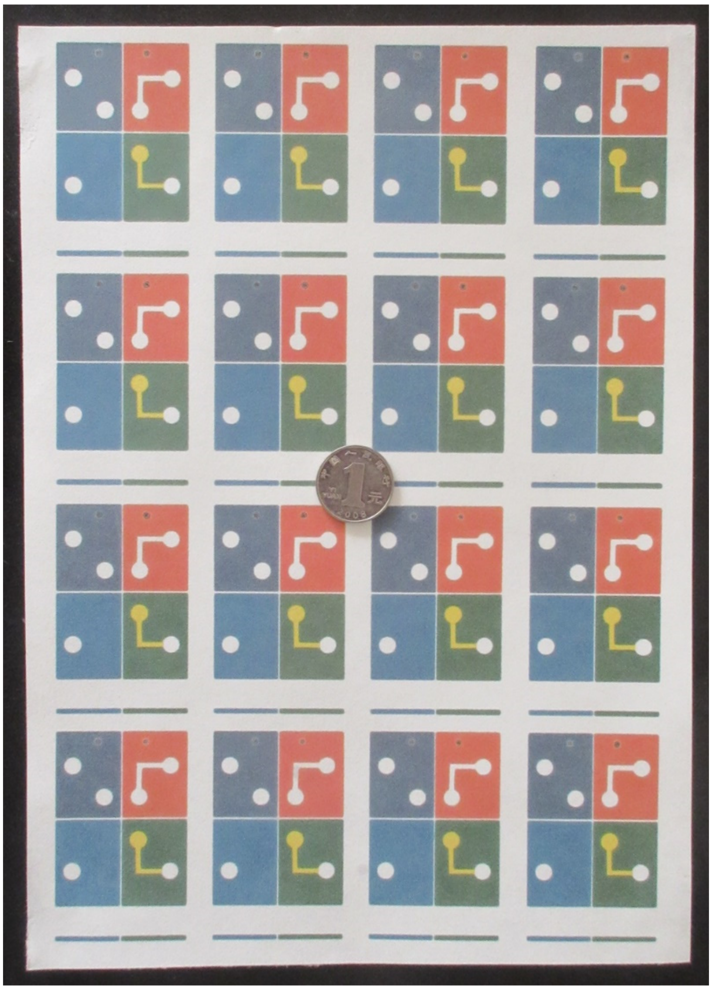

Figure S3. Right side of the baked wax-patterns of 3D-μ-MOBFCAD on a paper sheet (A4) after dropping CNT/Au on via hole.

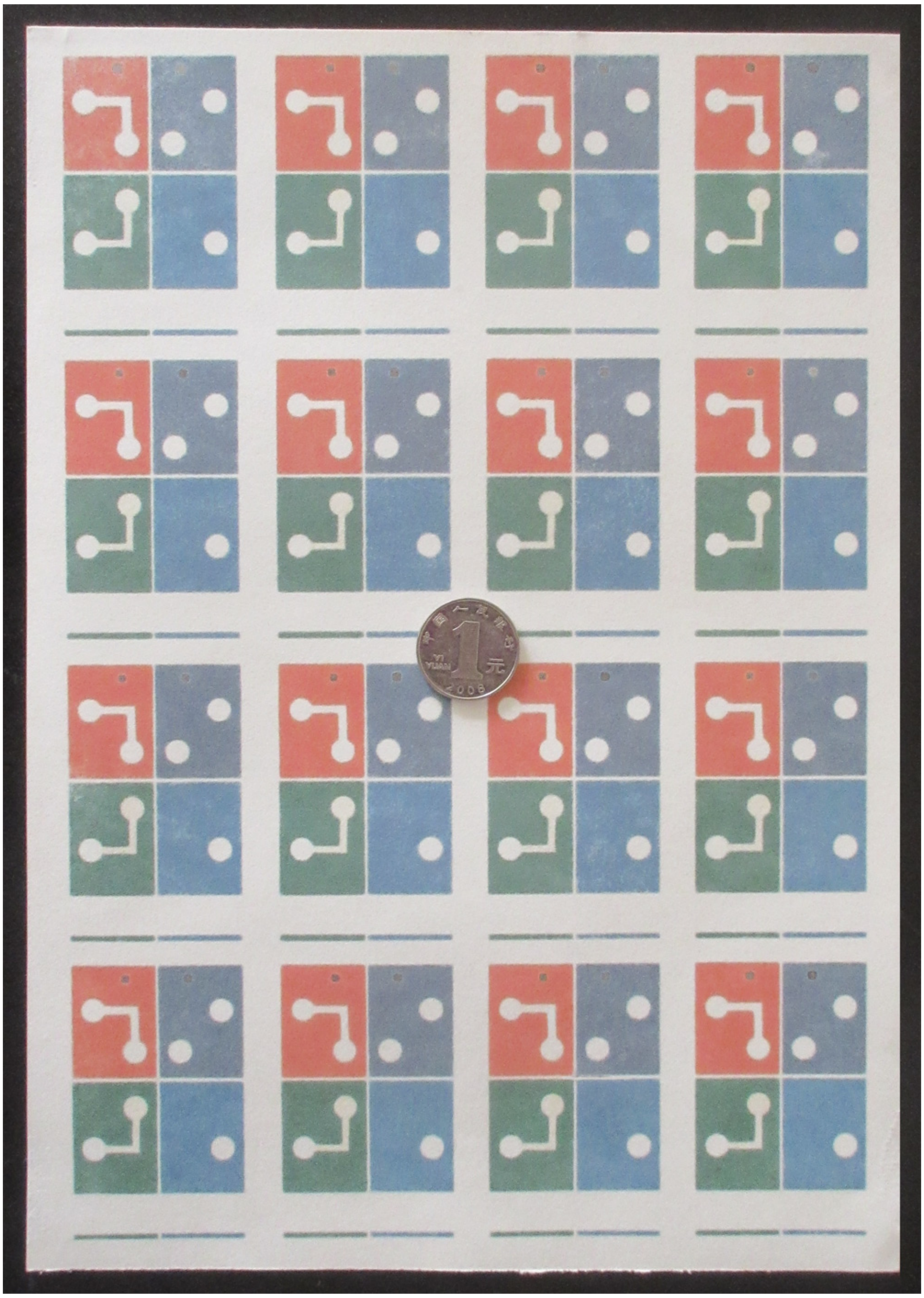

Figure S4. Reverse side of the baked wax-patterns of 3D-μ-MOBFCAD on a paper sheet (A4) after dropping CNT/Au on via hole.

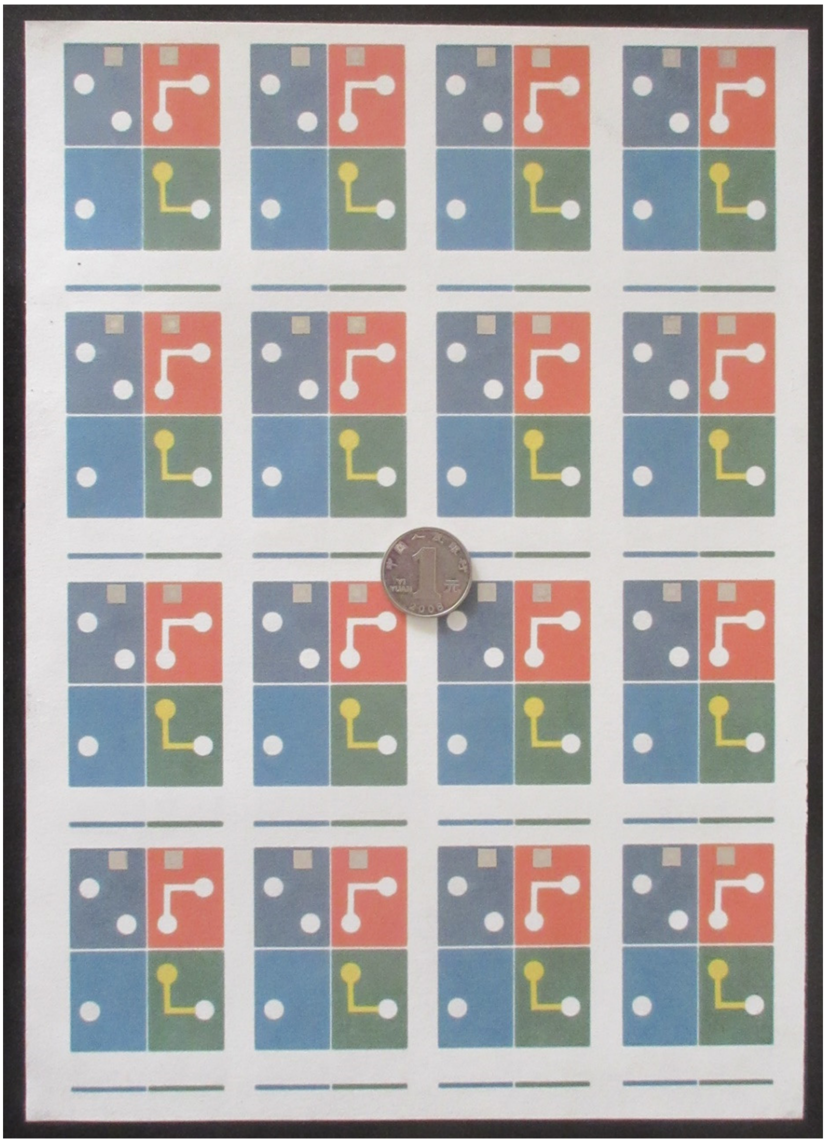

Figure S5. Right side of the 3D-μ-OBFCAD on a paper sheet (A4) after screen-printing of carbon electrodes, silver wire and drawing of graphite electrodes.

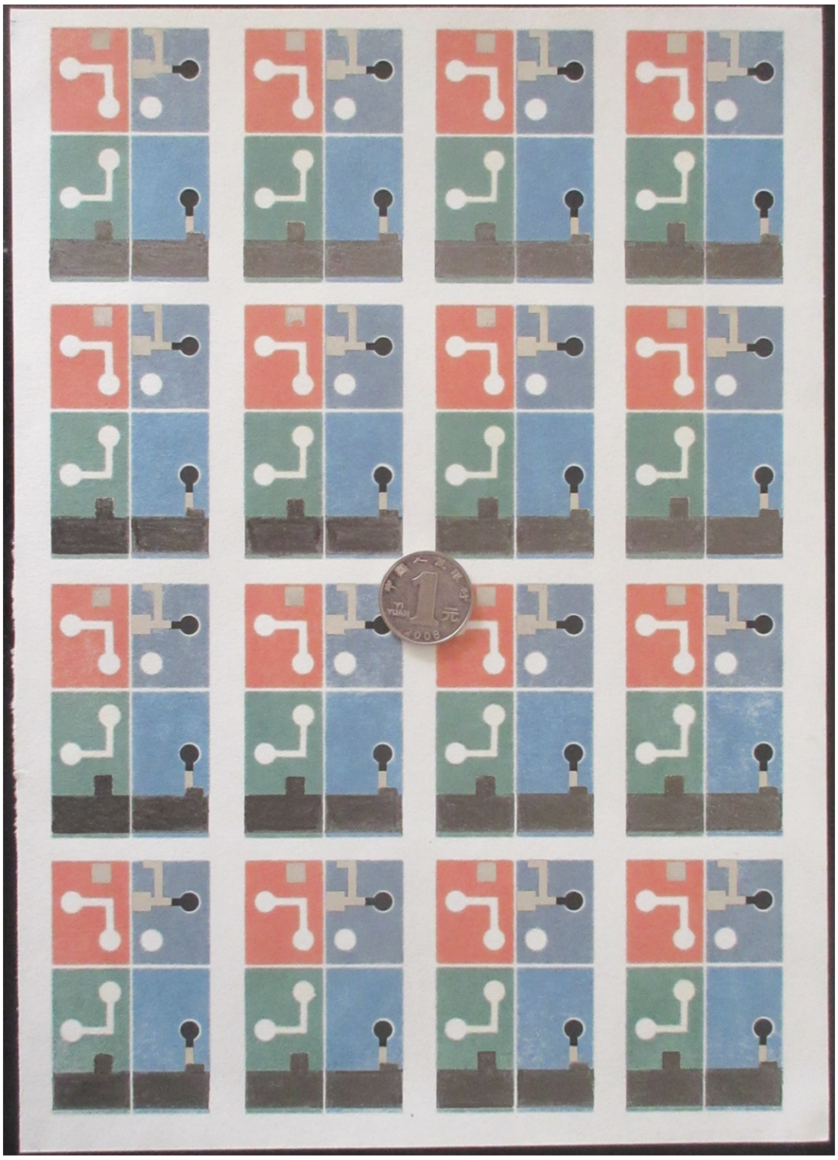

Figure S6. Reverse side of the 3D-μ-OBFCAD on a paper sheet (A4) after screen-printing of carbon electrodes, silver wire and drawing of graphite electrodes.

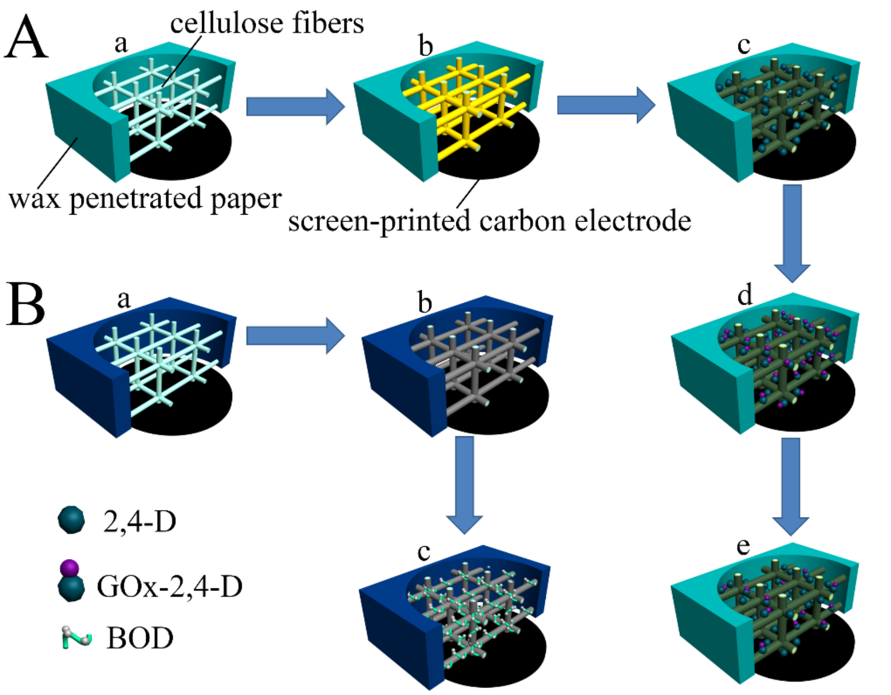

Scheme S1. Schematic diagram of the fabrication of bioanode (A) and biocathode (B), different part in (A) represents PAE (a), Au-PAE (b), MIPs-Au-PAE (c), GOx-2,4-D occupied MIPs-Au-PAE (d), and detection of 2,4-D at MIPs-Au-PAE; different part in (B) represents PCE (a), CNT-PCE (b), and BOD-CNT-PCE (c).

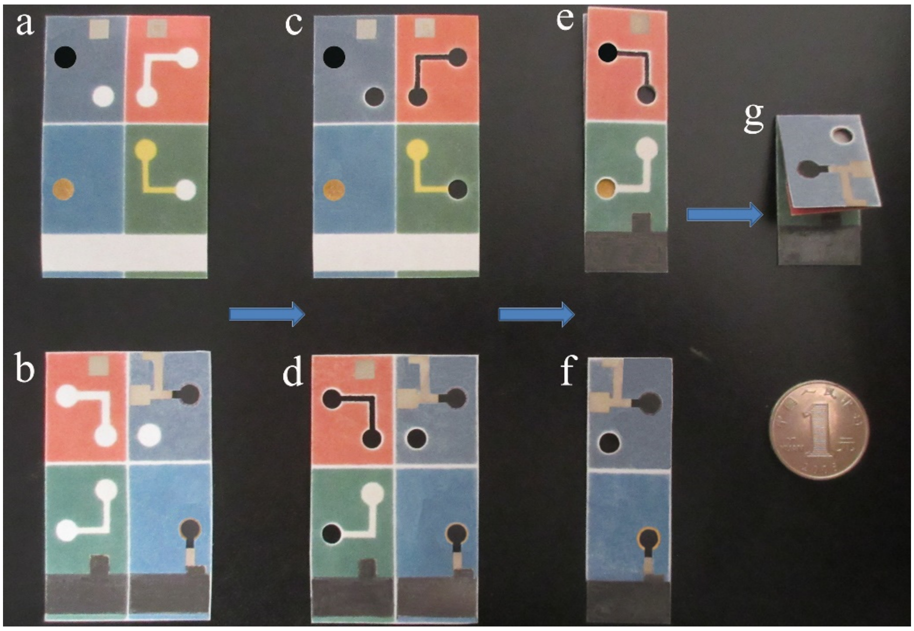

Figure S7. Schematic Representation, Size, Shape and Folding Procedure of the Wax Patterns on This 3D-μ-OBFCAD.

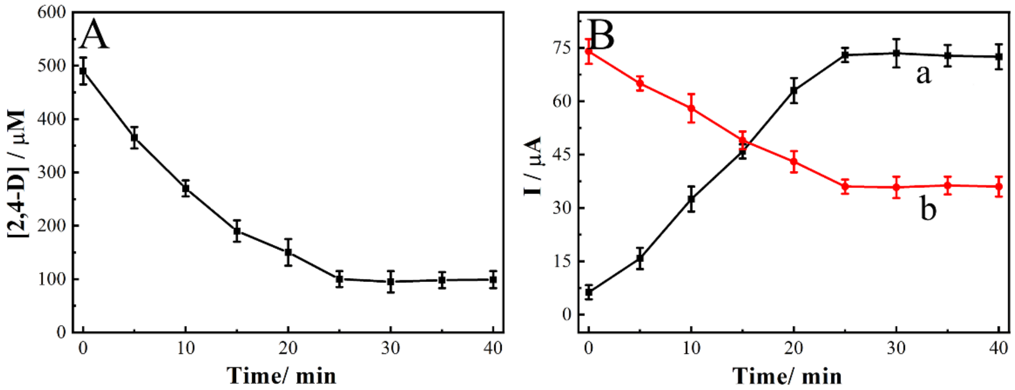

Figure S8. (A) Optimization conditions of blocking time in eluted MIP-grafted Au-PAE; (B) effects of (a) labeling time and (b) competition time in MIP-grafted Au-PAE.
